# Advances in CRISPR-Cas for Diagnosis and Treatment of *Klebsiella pneumoniae*

**DOI:** 10.3390/pathogens15060570

**Published:** 2026-05-26

**Authors:** Changmei Feng, Jun Yin

**Affiliations:** 1 Department of Clinical Laboratory, Sinopharm North Hospital, Baotou 014030, China; btfcm81@163.com; 2 College of Life Sciences, Inner Mongolia Agricultural University, Hohhot 010018, China

**Keywords:** antimicrobial resistance, CRISPR-Cas, gene editing, *Klebsiella pneumoniae*

## Abstract

Carbapenem-resistant *Klebsiella pneumoniae* (CRKP) is a significant pathogen for both hospital-acquired and community-acquired infections, characterized by its strong epidemic potential and high mortality rate, posing a severe threat to global public health. CRKP spreads widely across the globe through the horizontal transfer of plasmid-mediated resistance genes such as *blaKPC*, *blaNDM*, and *blaOXA-48*. The clinical treatment options for this bacterium are limited, and its resistance has been increasing year by year, urgently necessitating the development of new antimicrobial drugs or alternative strategies. In recent years, the CRISPR-Cas system has shown great potential in the diagnosis and treatment of CRKP, including rapid detection and identification, gene editing, antimicrobial strategies, and resistance inhibition. For instance, CRISPR-Cas12a/13a can be used for the rapid detection and identification of CRKP, while CRISPR-Cas9/Cas3 can target resistance genes to reverse the resistance of strains. With the advancement of delivery and biotechnologies, the CRISPR-Cas system is expected to become an important tool against drug-resistant CRKP. This review focuses on the application of the CRISPR-Cas system in the detection and treatment of CRKP, analyzing its technical advantages, limitations, and future development directions.

## Literature Search and Selection Strategy

This study systematically searched the PubMed database for relevant literature published between January 2010 and October 2025, covering the major developmental stages of CRISPR-Cas technologies, including Cas12, Cas13, Cas9, and Cas3, in the diagnosis and treatment of infectious diseases. The search strategy combined core keywords related to CRISPR-Cas systems, carbapenem-resistant *Klebsiella pneumoniae* (CRKP), as well as detection, diagnosis, therapy, and antimicrobial applications. Priority was given to studies involving clinical CRKP isolates, in vitro experiments, animal models, and preclinical evaluations. The inclusion criteria comprised English-language original research articles, reviews and case reports that provided experimental data on CRISPR-based diagnostic performance or antibacterial efficacy. Exclusion criteria included non-English publications, conference abstracts, non-peer-reviewed short communications, studies lacking valid CRKP-related data and studies focusing exclusively on other pathogens with limited applicability to CRKP. Literature analysis preferentially relied on studies directly involving CRKP strains, while findings derived from closely related members of t he Enterobacterales were interpreted with explicit acknowledgment of their limitations when extrapolated to CRKP. The literature screening process followed the PRISMA guidelines, involving stepwise selection through title and abstract screening followed by full-text review. Unpublished data and gray literature were excluded throughout the entire process.

## 1. Introduction

*Klebsiella pneumoniae* is a Gram-negative bacterium belonging to the genus *Klebsiella* in the family *Enterobacteriaceae.* It is a major pathogen responsible for both hospital- and community-acquired infections, particularly affecting hospitalized patients and immunocompromised individuals. It can cause pneumonia, urinary tract infections, bloodstream infections, liver abscesses, and other diseases [1]. Some strains of *Klebsiella pneumoniae* can lead to severe conditions such as pyogenic liver abscesses and meningitis, with high mortality rates. When it causes bloodstream infections, the mortality rate can reach up to 50% [2]. Carbapenem-resistant *Klebsiella pneumoniae* (CRKP) refers to *K. pneumoniae* strains that are resistant to carbapenem antibiotics, such as imipenem, meropenem, and ertapenem. CRKP is also one of the pathogens of *Enterococcus faecium*, *Staphylococcus aureus*, *Klebsiella pneumoniae*, *Acinetobacter baumannii*, *Pseudomonas aeruginosa*, and *Enterobacter* spp (ESKAPE). The World Health Organization has designated CRKP as a priority target for research and the development of new antibiotics [3]. Therefore, understanding the resistance mechanisms and transmission characteristics of CRKP is crucial for the targeted detection and treatment of antibiotic-resistant bacteria.

The CRISPR-Cas system, composed of clustered regularly interspaced short palindromic repeats (CRISPRs) and associated Cas proteins, serves as an adaptive immune mechanism in bacteria and archaea [4]. It protects against invading genetic elements such as phages and plasmids by capturing short sequences from foreign DNA—known as protospacers—and incorporating them into the host genome as spacers. This system is widely distributed, present in approximately 50% of bacterial genomes and 95% of archaeal genomes [5,6]. The immune response involves three key stages: adaptation, expression, and interference. Based on the structure and function of Cas proteins, the CRISPR-Cas system is classified into two major classes: Class 1 and Class 2, each further divided into three types [7]. Class 1 includes Type I, Type III, and Type IV, which use multi-protein complexes to cleave foreign DNA. Class 2 includes Type II (Cas9), Type V (Cas12), and Type VI (Cas13), typically utilizing a single Cas protein to cleave foreign DNA or RNA [6,8]. Some CRISPR-Cas systems target DNA (Type I, Type II, and most Type V), while others act by targeting RNA (Type III and Type VI) [9]. Due to its functional characteristics, the CRISPR-Cas system has extensive applications as a gene-editing tool. In the field of microbiology, it can be used for the rapid detection and treatment of antibiotic-resistant bacteria (Table 1).

## 2. The Drug Resistance Mechanisms of CRKP and Its Transmission Modes

### 2.1. The Drug Resistance Mechanisms of CRKP

The main drug resistance mechanism of *KP* to carbapenems is the production of carbapenemases, which are β-lactamases capable of hydrolyzing carbapenem antibiotics [16]. Based on their molecular structure, they can be classified into Class A, B, and D. Class A enzymes, such as *Klebsiella pneumoniae* carbapenemase (*KPC*), are currently the most common carbapenemases, mostly mediated by plasmids, and can spread rapidly through horizontal gene transfer. Class B enzymes, also known as metallo-β-lactamases (MBLs), include *NDM*, *VIM* and *IMP*. These enzymes require zinc ions as a catalytic center. Class D enzymes, such as *OXA-48*, have weaker hydrolytic activity against carbapenems but can still cause resistance [17]. The production of these enzymes is a major issue leading to difficulties in treating antibiotic-resistant bacteria.

In addition to carbapenemase production, *Klebsiella pneumoniae* can acquire resistance to carbapenem antibiotics through several other mechanisms. One such mechanism involves the loss or mutation of outer membrane proteins (OMPs), such as OmpK35 and OmpK36, which impede the entry of antibiotics into the bacterial cell, thereby contributing to resistance [18,19,20]. Another important mechanism is the efflux pump system, a membrane-bound complex that expels antibiotics from the bacterial interior to the external environment [21]. Overexpression of these pumps reduces intracellular antibiotic concentrations, leading to decreased susceptibility. Notably, several efflux pumps have been implicated in carbapenem-resistant *K. pneumoniae* (CRKP), including members of the *Resistance-Nodulation-Division* (RND) family, such as *tmexCD-toprJ* and *AcrAB-TolC* [22]. Additionally, chromosomal mutations can alter antibiotic targets, diminish drug binding efficiency, or modify bacterial metabolic pathways, thereby reducing antibiotic toxicity [2,23]. The formation of biofilms also contributes to resistance. These biofilms—polysaccharide-rich matrix structures secreted by the bacteria—act as physical barriers that protect the bacteria from environmental threats, including antibiotic exposure. For example, biofilms can hinder the penetration of carbapenem antibiotics, limiting drug-bacteria interactions and promoting resistance [24,25].

**Figure 1:**
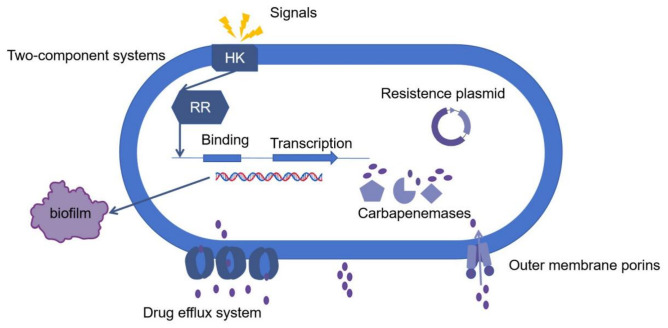
Mechanisms of Antibiotic Resistance in *Klebsiella pneumoniae.* HK, histidine kinase; RR, response regulator. Schematic illustration of the main antibiotic resistance mechanisms in *K.pneumoniae*, including two-component regulatory systems, carbapenemase production, efflux pump overexpression, outer membrane porin downregulation, and biofilm formation.

In summary, the mechanisms underlying carbapenem resistance in *K. pneumoniae* are multifaceted and often synergistic, significantly complicating clinical treatment and posing a serious challenge to infection management [26] (Figure 1).

### 2.2. Global Spread of CRKP

The development of resistance and global spread of CRKP is a complex global issue, with the widespread transmission of *KPC* enzymes being the primary mechanism for carbapenem resistance in *Klebsiella pneumoniae* worldwide. The gene encoding the *KPC* enzyme is typically located on mobile genetic elements (MGEs), such as plasmids or transposons, and resistance genes spread extensively within and between species via horizontal gene transfer, leading to global resistance [27,28,29]. Since the discovery of *KPC*-producing *Klebsiella pneumoniae* in 2001, more than 100 *KPC* variants have emerged, most of which result from gene mutations, deletions, insertions, or tandem repeats in *blaKPC-2* and *blaKPC-3*, such as new variants like *KPC-51* and *KPC-52*, which are resistant to ceftazidime/avibactam [30]. Due to the widespread ability of resistance genes to spread, the incidence of CRKP has dramatically increased. It has been reported that in 2021, the proportion of CRKP among *Klebsiella* species in China was between 20.9% and 24.2%, with more than 70% of these strains producing *KPC*-type carbapenemases, and *KPC-2* being the dominant subtype [31]. The widespread spread of CRKP has led to the emergence of resistant strains in multiple countries around the world, with the Asia-Pacific region being a high-incidence area for CRKP. Related studies have investigated the prevalence of CRKP resistance across various countries, finding that CRKP resistance occupies a high proportion in each, such as 67% in Greece, 65% in Iran, 64% in Russia, 57% in India, 50% in Saudi Arabia, 45% in Peru, 33% in Italy, 26% in Argentina, 24% in Brazil, and 11% in the United States [31,32].

In addition to *KPC* enzymes, *NDM* and *OXA-48* enzymes are also major carbapenemases that contribute significantly to the global dissemination of CRKP. New Delhi metallo-β-lactamase (*NDM*), a class B metallo-β-lactamase, is capable of hydrolyzing nearly all β-lactam antibiotics, including carbapenems [33,34]. The gene encoding *NDM*, *blaNDM* is typically located on mobile plasmids, such as the IncX3-type, which possess a broad host range and facilitate horizontal gene transfer across different bacterial species, contributing to widespread global transmission. Since the initial identification of *NDM-1* in 2008 in New Delhi, India, *NDM*-type enzymes have rapidly spread worldwide. In regions such as India, Pakistan, and Bangladesh, *NDM* enzymes are now the predominant mechanism of resistance among carbapenem-resistant Enterobacterales (CRE), posing a major challenge to clinical treatment by significantly reducing the effectiveness of last-resort antibiotics [35]. *OXA-48*, a class D carbapenemase, is another critical resistance factor, typically carried on IncL-type plasmids. It is currently the second most common carbapenemase in Enterobacterales in India, after *NDM*. *OXA-48*-producing strains are notoriously difficult to detect in standard clinical laboratories, often leading to underdiagnosis, ineffective infection control, and the silent spread of resistance [15]. This diagnostic challenge contributes to the underestimation of *OXA-48*’s clinical impact and hinders timely therapeutic intervention [36]. Given the rapid global emergence of CRKP and its association with multiple potent carbapenemases, there is an urgent need to strengthen surveillance, slow the spread of resistance, and improve therapeutic strategies to combat CRKP and protect global public health.

Clinically, the treatment of CRKP faces significant challenges, as most conventional antibiotics are ineffective against it. Polymyxin and tigecycline are often regarded as the “last line of defense” [37]. Novel β-lactam/β-lactamase inhibitors, such as ceftazidime/avibactam, are also important drugs for treating CRKP [38,39]. However, due to improper infection control and antibiotic management in clinical settings, resistance to these drugs has been increasing year by year [40,41]. Therefore, the search for new antibiotics and treatment strategies has become urgent. In recent years, with the rise of CRISPR-Cas gene editing research, its role in CRKP detection and treatment has garnered widespread attention, and in the future, the application of the CRISPR-Cas system in clinical antibiotic-resistant bacteria may be achievable.

## 3. Application of CRISPR-Cas in the Detection of CRKP

### 3.1. The Basic Mechanism of CRISPR-Cas Detection

**Figure 2:**
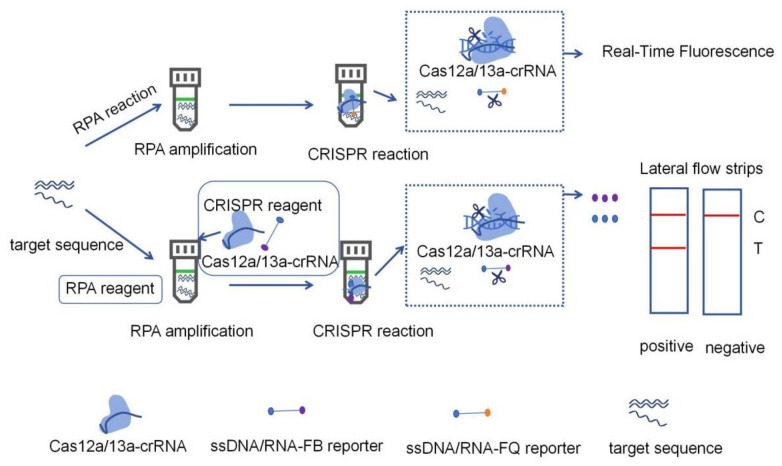
Application of CRISPR-Cas in the detection of CRKP. Abbreviations: CRKP, carbapenem-resistant *Klebsiella pneumoniae*; RPA, recombinase polymerase amplification; CRISPR, clustered regularly interspaced short palindromic repeats; crRNA, CRISPR RNA; ssDNA/RNA-FB reporter, single-stranded DNA/RNA fluorescence-biotin reporter; ssDNA/RNA-FQ reporter, single-stranded DNA/RNA fluorescence-quenching reporter; C, control line; T, test line. This schematic illustrates two common CRISPR-Cas-based workflows for nucleic acid detection: (1) pre-amplification of target sequences by RPA followed by CRISPR-mediated cleavage and real-time fluorescence readout; (2) one-pot RPA-CRISPR reactions with either fluorescence-based or lateral flow strip-based visual detection.

Accurate species identification is critical for clinical diagnosis and treatment. While traditional molecular biology identification methods (e.g., PCR) are time-consuming and complex, the CRISPR-Cas system provides a rapid and accurate alternative. By designing specific sgRNAs, the CRISPR-Cas system can specifically recognize and cleave specific gene sequences of CRKPs, enabling rapid species identification [42]. The core of the CRISPR-Cas system consists of Cas proteins and guide RNA (gRNA). The gRNA can be designed to target conserved sequences or resistance genes in the *Klebsiella pneumoniae* genome. When the Cas protein binds to the gRNA, it forms a complex that recognizes and binds to the target gene sequence, followed by cleavage of the target gene by the Cas protein [43]. Currently, according to the nucleic acid detection principles of the CRISPR-Cas system, there are two types: one utilizes the properties of Cas proteins such as Cas9 to specifically recognize and bind dsDNA, while the other uses the characteristics of Cas13, Cas12 and other Cas proteins to activate trans-cleavage activity after specific nucleic acid recognition, cleaving ssDNA or ssRNA nonspecifically [9]. By combining highly sensitive biosensing technologies to detect the cleavage-marked single-stranded DNA or RNA, rapid, sensitive, and specific detection of *K. pneumoniae* can be achieved. For example, the CRISPR-Cas12 and Cas13 systems can activate both cis and trans cleavage activities upon recognizing target DNA or RNA, leading to the cleavage of fluorescent probes and signal amplification for detection (Figure 2) [43]. Studies have shown that this detection method has extremely high sensitivity and specificity, making it suitable for the rapid identification of resistant genes and strain typing (Table 2).

The primary mechanism of carbapenem resistance in CRKP is the production of carbapenemase enzymes [17]. These enzymes differ in their resistance profiles and biochemical characteristics, necessitating targeted detection and treatment strategies. Accurate identification of the specific carbapenemase involved is therefore crucial for guiding effective clinical management. CRISPR-Cas technology offers a powerful tool for the precise detection of carbapenemase genes through the design of specific single-guide RNAs (sgRNAs) [44,45]. For instance, sgRNAs targeting the *KPC* or *NDM* genes can rapidly and accurately detect the presence of the corresponding enzymes. This gene-specific detection enables rapid identification of resistance mechanisms and supports precision antimicrobial therapy by informing timely and appropriate antibiotic selection.

### 3.2. Detection Based on CRISPR-Cas12/13

#### 3.2.1. CRISPR-Cas12

Cas12 is a Class V nucleic acid endonuclease, a single-stranded DNA and double-stranded DNA endonuclease guided by crRNA. The Cas12a protein, also known as Cpf1, can independently process precursor crRNA without the need for tracrRNA or RNaseIII. Its protospacer adjacent motif (PAM) sequence is usually 5′-TTTN-3′ or 5′-TTN-3′, which can generate sticky ends, facilitating DNA insertion and replacement. Under the guidance of crRNA, the PAM site, rich in thymine, can activate CRISPR-Cas12a for targeted dsDNA cleavage (cis-cleavage) and can also activate CRISPR-Cas12a for non-targeted cleavage (trans-cleavage). Currently, the trans-cleavage activity of Cas12a has become an ideal tool for nucleic acid and pathogen detection. The DETECTR detection platform, built on this, has been successfully applied in various clinical and molecular diagnostics [9,10,46,47,48].

In *Klebsiella pneumoniae* detection, CRISPR-Cas12a is often combined with isothermal amplification techniques for the detection of specific genes and carbapenem resistance genes [49]. Yang combined Recombinase Polymerase Amplification (RPA) with CRISPR-Cas12a to build the RCCS platform, which can detect carbapenemase genes *KPC* and *NDM* in bacteria within 50 min. The sensitivity for *KPC* reaches 10 ng/μL, while for *NDM*, it reaches 1 ng/μL, with 100% accuracy confirmed by isolate strain identification. This platform visualizes detection results using lateral flow test strips and fluorescence technology [50]. Tan constructed a dual RPA and CRISPR-Cas12a platform for simultaneous detection of rcsA and *KPC* genes in *KP*, achieving sensitivities of 1 × 10^1^ pg/μL and 1 × 10^2^ pg/μL, with accuracy rates of 100% and 94.92%, respectively, when compared to culture and antibiotic susceptibility methods [51]. Both LAMP and RPA can be combined with CRISPR-Cas12a to construct DETECTR platforms. To achieve higher sensitivity and accuracy, Li et al. compared these methods and found that RPA-CRISPR-Cas12a was the most effective for detecting *KPC* [44].

Based on the CRISPR-Cas12a system, researchers have developed a combined RPA–CRISPR-Cas detection system that enables rapid amplification and identification of target nucleic acids at a constant temperature. Compared to traditional molecular biology techniques or culture-based methods, this CRISPR-Cas-based detection approach offers significantly reduced turnaround times and simplifies operational requirements. Its minimal reliance on complex instrumentation enhances both practicality and cost-effectiveness, making it especially suitable for point-of-care testing and resource-limited settings. Furthermore, the CRISPR-Cas system broadens the applicability of diagnostic methods by offering diverse result readout formats, such as fluorescence detection and lateral flow strip assays, which improve both accuracy and ease of use. These platforms operate faster than conventional quantitative PCR (qPCR), allowing for a substantial reduction in the diagnostic cycle and enabling more timely clinical decision-making [10,44,46]. In addition, the CRISPR-Cas system supports multiplex detection, allowing for the simultaneous identification of multiple genetic targets. This capability is particularly advantageous in the study of complex gene networks and multi-drug resistance, where rapid and comprehensive genetic profiling is essential [51]. Overall, CRISPR-Cas-based diagnostics provide a powerful and flexible solution for improving the speed, accuracy, and accessibility of clinical detection and antimicrobial resistance monitoring.

#### 3.2.2. CRISPR-Cas13

Cas13 (C2c2) is a Class VI nucleic acid endonuclease of the CRISPR-Cas system. Unlike Cas9 and Cas12, which target DNA, Cas13 targets single-stranded RNA (ssRNA) and possesses unique RNA editing and detection capabilities. Guided by crRNA, Cas13 specifically recognizes and cleaves target RNA. Upon activation, in addition to cleaving the target RNA, it also non-specifically cleaves surrounding RNA molecules, a property known as “collateral cleavage” or “trans-cleavage” [11,52]. Based on this characteristic, Cas13a has been used to construct the SHERLOCK platform, which shows great advantages in virus detection [53,54]. Furthermore, it can also be used for detecting resistance genes, such as 16S rRNA and carbapenemase resistance genes in *K. pneumoniae*.

In *K. pneumoniae*, Cas13a can recognize and cleave specific resistance genes. Cao combined polymerase chain reaction (PCR) and recombinase-assisted amplification (RAA) technologies with the CRISPR-Cas13a system to detect *KP* genes, as well as *KPC* and *NDM* resistance genes. The results showed that the detection platform had a sensitivity of 92.16% for detecting *K. pneumoniae* strains, with 100% accuracy and detection limits (LOD) of 1 cfu/cm^2^ and 10^1^ copies/μL, respectively [45]. Liang developed an RPA-Cas13a platform, which achieved a sensitivity of 96.5% for detecting *KPC* resistance plasmids in carbapenem-resistant Enterobacteriaceae, with a detection limit for recombinant plasmids containing the gene of 2.5 copies/μL [55]. Results from LAMP-Cas13a-based detection of the carbapenemase *OXA-48* and *GES-2* genes showed that the platform achieved 100% accuracy, with detection limits for *OXA-48* and *GES-2* resistance genes in *K. pneumoniae* at 10^3^ cfu/mL [56]. Both CRISPR-Cas12 and CRISPR-Cas13 systems can realize rapid and accurate diagnosis and detection of *K. pneumoniae* and its drug-resistant genes in a short period of time, and both of them are excellent in terms of specificity and accuracy. However, compared with the CRISPR-Cas12 system, the CRISPR-Cas13 system targets RNA, which not only makes it more advantageous in the field of transcriptome analysis and viral detection, but also poses a higher off-target risk [11,56,57]. Therefore, in the clinical application of CRISPR-Cas13 technology for the identification and detection of *K. pneumoniae* and its drug-resistant genes, special attention needs to be paid to its potential off-target effect, and the design of sgRNA and experimental conditions need to be optimized more precisely to ensure the accuracy and reliability of the detection.

Studies have shown that the CRISPR-Cas13 system is highly effective not only in characterizing and detecting microorganisms, but also in discriminating single-nucleotide variations, demonstrating exceptional sensitivity and specificity. For instance, the SHERLOCK v2 platform, developed by Gootenberg, leverages Cas13 for simultaneous detection of up to four distinct viruses or genetic mutations, showcasing powerful multiplexing capabilities [58]. Building on this, the Combinatorial Array Reaction for Multiplexed Evaluation of Nucleic acids (CARMEN) platform, developed by Ackerman et al. in conjunction with Cas13, allows for the simultaneous detection of more than 4500 crRNA targets with high robustness and scalability. This advanced system has enabled not only comprehensive subtyping of influenza A strains during the COVID-19 pandemic, but also multiplex identification of dozens of *HIV* drug-resistance mutations in a single assay [59]. These innovations highlight the remarkable versatility and precision of CRISPR-Cas systems in viral diagnostics [60]. Although direct reports of drug resistance due to point mutations in *K. pneumoniae* remain limited, similar CRISPR-Cas13-based approaches may be adapted to specifically detect resistance-conferring mutations by designing crRNAs that target relevant single-nucleotide polymorphisms. This holds promise for enhancing the precision of antibiotic resistance surveillance and informing more targeted treatment strategies in clinical settings.

### 3.3. Other CRISPR-Cas

In addition to Cas12a and Cas13a, which are widely used for *K. pneumoniae* detection, a new nuclease, Cas14, was discovered by Harrington in 2018 [61]. Compared to other Cas proteins, it has some unique advantages, such as being half the size of other proteins and not requiring PAM for recognizing ssDNA. The trans-cleavage activity activated by the target ssDNA gives Cas14a the ability to non-specifically cleave ssDNA gene side branches, providing an effective method for signal amplification [62,63]. Although there is no research yet showing the application of Cas14a in *K. pneumoniae* detection, many studies have demonstrated its application in detecting other bacteria and viruses, with results showing high sensitivity, specificity, and accuracy [12,57,63,64]. Based on this, it is speculated that Cas14a may become an important gene editing tool for *Klebsiella pneumoniae* detection in the future.

## 4. Application of CRISPR-Cas in the Treatment of CRKP

**Figure 3:**
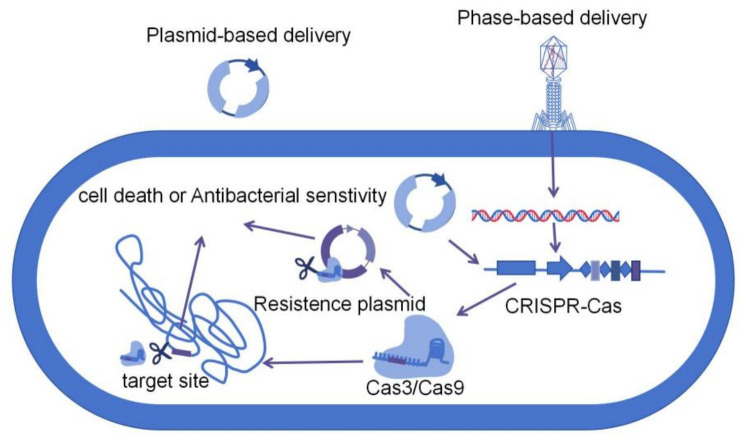
Application of CRISPR-Cas in the treatment of CRKP.

Due to the strong resistance of CRKP and the limited effectiveness of traditional antibiotic treatments, CRISPR provides the possibility of specifically targeting and eliminating resistant bacteria. The treatment strategy of the CRISPR-Cas system for resistant strains generally involves targeting resistance genes, activating specific sequences, or eliminating resistance plasmids through precise gene editing. This technology operates through spacer sequences, PAM sequences, and Cas proteins, enabling precise interference at specific chromosomal or plasmid sites [7]. It offers new ideas for the development of novel antimicrobial strategies and shows great potential in the detection, treatment, and prevention of resistant bacteria (Figure 3, Table 3).

### 4.1. CRISPR-Cas9

CRISPR-Cas9 was the first system applied to gene editing and enables precise genetic modifications by specifically recognizing and cleaving target DNA sequences [65]. The system consists of CRISPR RNA (crRNA), trans-activating crRNA (tracrRNA), and the Cas9 protein. The crRNA guides the system to the target DNA sequence through a ~20-nucleotide guide region at its 5′ end, while its 3′ end contains a repeat sequence that binds to the tracrRNA. The crRNA and tracrRNA together form a double-stranded RNA complex known as the single-guide RNA (sgRNA), which activates the Cas9 protein [9]. Cas9 is the core nuclease of the CRISPR-Cas9 system and is classified as a type II CRISPR-associated endonuclease. It binds to the sgRNA and facilitates site-specific cleavage of double-stranded DNA (dsDNA). Cas9 contains two nuclease domains, HNH and RuvC, each of which cleaves one strand of the DNA, resulting in a double-strand break (DSB) [6,13]. For Cas9 to function, it must first recognize a protospacer adjacent motif (PAM) sequence on the target DNA. This recognition ensures the specificity of crRNA binding and activates Cas9’s nuclease activity. Following cleavage, the DSB is primarily repaired via two pathways: non-homologous end joining (NHEJ) and homology-directed repair (HDR). NHEJ directly ligates the broken DNA ends, often introducing insertions or deletions (indels). In contrast, HDR uses a homologous DNA template, such as donor DNA, to enable precise gene correction or insertion [13]. Most bacteria lack the non-homologous end joining (NHEJ) pathway and instead rely on exogenous donor DNA templates to repair double-strand breaks via the homology-directed repair (HDR) pathway. This mechanism enables gene editing through the CRISPR-Cas system by designing crRNAs that specifically target one or more genes, allowing for precise genetic modifications [66]. The widely used CRISPR-Cas9 system consists of two main components: the Streptococcus pyogenes Cas9 nuclease and a single-guide RNA (sgRNA), which is an artificially engineered chimeric RNA combining crRNA and tracrRNA [67,68].

*K. pneumoniae* focuses on delivering the Cas9 system into bacteria through phages or plasmids to achieve the cleavage of drug-resistant genes for treatment. Wang constructed a single plasmid system, pCas9-sgRNAKP, and introduced it into *K. pneumoniae*, finding that the strains were effectively killed and that the Cas9 system had therapeutic effects on the bacteria. To further investigate the resistance mechanisms of CRKP, they also constructed a dual-plasmid genomic editing system, pCasKP-pSGKP, and a single-plasmid base editing system, pBECKP. They found that plasmids carrying the *KPC-2* and *CTX-M-65* resistance genes were directly inactivated or cleared under the system’s action without repair, affecting the spread of bacterial resistance [67]. Ding knocked out *NDM-1* using Cas9 and found that after the *NDM-1* gene was knocked out, CRKP regained sensitivity to imipenem, antimicrobial peptides, and the combination of sulbactam in both in vitro and in vivo experiments [34]. Hao used the plasmid curing system pCasCure to demonstrate that CRISPR-Cas9-mediated *KPC* and *OXA-48* gene editing, as well as plasmid curing, made CRKP sensitive again to carbapenem antibiotics [69]. Clinically, tigecycline and polymyxin are often considered the “last line of defense” for CRKP treatment, but in recent years, resistance to polymyxin has been increasing. CRISPR-Cas gene editing may become an auxiliary therapeutic strategy for treating polymyxin resistance [40]. Sun knocked out the *tetA*, *ramR* and *mgrB* genes and found that Cas9 knockout of the *mgrB* gene significantly increased the MIC of polymyxin, while knockout of the *tetA* gene significantly increased bacterial sensitivity to tigecycline. On the other hand, knockout of the *ramR* gene led to a sharp increase in resistance to tigecycline [70]. Research has shown that in polymyxin-resistant CRKP strains, knocking out *CcrB* using a CRISPR-Cas9/lambdared recombinant gene editing system resulted in a reduction of the MIC for polymyxin from 64 μg/mL to 2 μg/mL [71]. Sun found that knocking out the lipid A palmitoyltransferase (*pagP*) gene, which assists in the bacterial outer membrane barrier, helped in treating CRKP [72]. High-virulence carbapenem-resistant *Klebsiella pneumoniae* (Hv-CRKP) is an even more critical clinical issue, with *clpV* and *rmpA* being key virulence-related genes. Liu tracked the nosocomial transmission of Hv-CRKP and discovered *rmpA* variants. After knocking out *rmpA* using Cas9, they found that the active PT11-rmpA regulatory factor was a biomarker of the highly virulent ST11-K64/CRKP clone [73]. They also found that knocking out the virulence-related gene *clpV* did not affect the growth of *K. pneumoniae* but reduced its hypermucoviscosity phenotype and biofilm formation, decreasing its competitiveness with other bacteria [74]. Experimental studies have demonstrated that the CRISPR-Cas9 gene editing system, when delivered into bacteria via vectors such as plasmids, can precisely target and cleave genes responsible for antibiotic resistance and virulence. This targeted gene disruption not only restored bacterial susceptibility to carbapenem antibiotics but also significantly reduced the virulence and competitive fitness of drug-resistant strains, thereby enhancing therapeutic efficacy. These findings strongly support the potential of the CRISPR-Cas9 system as an effective tool for combating drug-resistant bacteria. However, most of these studies have been limited to in vitro settings, with relatively few investigations evaluating the therapeutic effects of CRISPR-Cas9 against CRKP in in vivo models. Although no direct in vivo studies targeting CRKP with CRISPR-Cas9 have been reported to date, related research has shown promising outcomes in other pathogens. For example, the CRISPR-Cas9 system has been successfully used to reduce colonization of methicillin-resistant *Staphylococcus aureus* (MRSA) in a mouse skin infection model [75]. Furthermore, CRISPR-Cas9-modified bacteriophages, delivered via alginate hydrogels, significantly reduced soft tissue infections in MRSA-induced rat models of osteomyelitis and soft tissue infection. Remarkably, the therapeutic effect was comparable to that of high-dose fosfomycin [76]. These in vivo results underscore the feasibility and translational potential of the CRISPR-Cas9 system for treating infections caused by drug-resistant bacteria, and they provide a valuable reference point for future development of CRISPR-based therapies targeting CRKP. Despite its promising potential, achieving effective in vivo treatment of infections using the CRISPR-Cas9 system remains challenging. One of the primary obstacles lies in the need to further optimize in vivo delivery strategies to enhance delivery efficiency and ensure precise targeting of infectious pathogens. Successfully directing the CRISPR-Cas9 system to drug-resistant bacteria within the host environment is essential for effective bacterial clearance. Moreover, in most prokaryotic organisms, CRISPR-Cas9—being a heterologous and relatively large gene-editing system—faces additional barriers. Its expression can be significantly impaired by the complex, dynamic, and often unstable intracellular environment in microbial cells, particularly due to the tight regulation of DNA homeostasis. These challenges limit its applicability across various prokaryotic species and highlight the need for further engineering of more compact, efficient, and host-adapted CRISPR systems for broader therapeutic use.

### 4.2. CRISPR-Cas3

The Type I CRISPR-Cas system is the most prevalent CRISPR-Cas system found in nature and is widely distributed among various bacteria, including *K. pneumoniae*, where subtypes such as I-E and I-E* have been identified [77]. Unlike the more commonly known Type II CRISPR-Cas system, the Type I system is capable of endogenous genetic engineering through self-targeting mechanisms. CRISPR-Cas3, a key tool within the Type I system, stands out for its ability to mediate large-fragment deletions with high precision and low off-target effects [78]. These features make it particularly promising for applications in gene therapy and genome editing, especially for targeting drug-resistant gene clusters or virulence-associated genes. Cas3 operates by being guided to the target DNA via a CRISPR RNA (crRNA), where it binds and initiates DNA degradation from the recognition site using its 3′-to-5′ exonuclease activity. Unlike Cas9, which typically introduces double-strand breaks, Cas3 not only cuts DNA but continues to degrade it, enabling efficient removal of large genomic regions. Despite its powerful capabilities, the CRISPR-Cas3 system is more complex than its Type II counterpart. Its function depends on a multisubunit complex, such as Cascade, to recognize and bind the target DNA. Moreover, Cas3-mediated degradation is unidirectional, proceeding primarily upstream of the PAM (Protospacer Adjacent Motif) sequence. This directional activity allows for more controlled and precise gene editing, minimizing unintended DNA damage [14].

In *K. pneumoniae*, subtype I-E and I-E* CRISPR-Cas systems are negatively associated with antimicrobial resistance and can inhibit the spread of IncF plasmids carrying the *KPC-2* resistance gene [28,79,80,81]. In addition, Kamruzzaman also discovered a plasmid IV type CRISPR-Cas system in *Klebsiella* species, which carries multiple β-lactamase genes, including *CTX-M*, *NDM* and *OXA*. They speculated that the plasmid IV-type CRISPR may depend on the functionality of the chromosomal I-E CRISPR system [79]. However, recent studies have shown that type I CRISPR-Cas systems can coexist with the *blaKPC* gene on the same plasmid. For instance, in *K. pneumoniae* sequence type ST15, the *blaKPC*-IncFII plasmid and the subtype I-E CRISPR-Cas system have been found to coexist [82]. This phenomenon may be attributed to the presence of an anti-CRISPR protein (Acr). Wang et al. identified the anti-CRISPR protein AcrIE9.2 in ST15 strains, which inhibits the activity of the I-E CRISPR-Cas system [83]. This suppression prevents the system from targeting the *blaKPC*-IncFII plasmid, thereby allowing its stable acquisition and maintenance. These findings indirectly highlight the role of CRISPR-Cas systems in restricting the dissemination of antibiotic resistance genes and offer a theoretical foundation for their potential application as endogenous gene-editing tools. Building on these findings, Zhou et al. developed a plasmid immunization platform based on the endogenous type I CRISPR-Cas3 system of *K. pneumoniae*. They engineered the pCRISPR platform by integrating the CRISPR-Cas3 system into a self-replicating, conjugative plasmid vector. This system demonstrated high efficiency in eliminating IncFII-type drug-resistant plasmids, thereby re-sensitizing resistant *K. pneumoniae* strains to antibiotics. Both in vitro and in vivo experiments showed that the CRISPR-Cas3 system could remove up to 90% of IncFII plasmids. In an in vivo infection model using Galleria mellonella larvae, the CRISPR-Cas3 system not only eliminated the resistance plasmids but also reduced bacterial virulence and significantly improved host survival rates [77]. These findings highlight the strong potential of the native type I CRISPR-Cas3 system as an effective and feasible therapeutic tool against multidrug-resistant bacteria. By targeting conserved regions of plasmids, the CRISPR-Cas3 system offers a novel strategy to eliminate resistance genes and restore antibiotic susceptibility in pathogenic strains. Moreover, beyond *K. pneumoniae*, other naturally encoded CRISPR-Cas systems in prokaryotes are increasingly being recognized as powerful tools for genetic manipulation. Therefore, further exploration and development of natural CRISPR-Cas systems hold great promise for clinical interventions against drug-resistant bacterial infections.

**Table 3:** Applications of the CRISPR-Cas System in the Treatment of CRKP.

CRISPR-Cas	Delivery Method	Introduction Method	Knocked-Out Gene	References
Cas9	pCasKP-pSGKP	Electroporation	*KPC-2*, *CTX-M-65*	[67]
Cas9	pX458	CaCL_2_ transformation	*NDM-1*	[34]
Cas9	pCasKP-pSGKP	Electroporation	*tetA*, *ramR*, *mgrB*	[70]
Cas9	pCasCure	Electroporation	*KPC-2*, *OXA-48*	[69]
Cas9	pCas9-lambda red	Electroporation	*CrrB*	[71]
Cas9	pCasKP-pSGKP	Electroporation	*pagP*	[72]
Cas9	pCasKP-pSGKP	Heat activation	*rmpA*	[73]
Cas9	pCasKP-pSGKP	Electroporation	*clpV*	[74]
Cas3	pCRISPR	Electroporation, Conjugative transfer	lncFII	[77]
CRISPRi	pdCas9gRNA	Electroporation	*NDM-1*, *SHV-12*	[78]
CRISPRi	pJMP1363	Electroporation	*OXA-48*	[15]

Abbreviations: The elements indicated in italics in the table are genes; pCasKP-pSGKP: A dual plasmid system for genome editing of Klebsiella pneumoniae (Klebsiella pneumoniae). It combines the CRISPR/Cas9 gene editing system and the λ-Red homologous recombination system; pX458: Plasmid vectors for gene editing research; pCasCure, pCRISPR: A plasmid based on the CRISPR-Cas9 system; pCas9-lambda red: A plasmid combining the CRISPR/Cas9 gene editing system and the λ-Red homologous recombination system; pdCas9gRNA: A plasmid incorporating the CRISPRi system; pJMP1363: A plasmid vector for the CRISPRi system.

### 4.3. CRISPRi

CRISPR interference (CRISPRi) differs from traditional gene editing technologies in that it does not cut DNA but instead inhibits gene expression by physically blocking the binding or movement of RNA polymerase. Its mechanism involves using an inactivated Cas protein (such as dCas9) to bind to the promoter region or transcription start site of the target gene, thereby inhibiting gene transcription without causing double-strand breaks or relying on homology-directed repair. It is a gene expression regulation technology derived from the type II CRISPR system [84]. This system has been widely used to study gene function and has shown significant advantages in discovering resistance targets, exploring pathogenicity and virulence, analyzing bacterial cell growth phenotypes, enhancing bacterial metabolites, and studying genetic interactions [85,86,87,88].

CRISPRi technology in the treatment of *K. pneumoniae* is reflected in the targeted inhibition of specific gene expression and the identification of genes related to bacterial resistance and virulence. Researchers like Yao developed an integrated CRISPRi system that can effectively silence the expression of single and multiple resistance genes in the plasmids of CRKP, such as *NDM-1*, *SHV-12* and others. Studies have shown that inhibiting the single resistance gene *NDM-1* reduced the MIC of meropenem by more than 1000 times. At the same time, silencing both the *NDM-1* and *SHV-12* resistance genes led to a 16-fold and 8-fold decrease in the MIC of meropenem and imipenem, respectively [85]. CRISPRi can also inhibit the *OXA-48* carbapenemase resistance gene in *K. pneumoniae*, reducing the MIC of meropenem from 64 mg/L to 16 mg/L [15]. These results indicate that CRISPRi can show significant advantages in the treatment of CRKP. Furthermore, CRISPRi can also be used to construct gene knockdown libraries for *Klebsiella pneumoniae* to screen for functional genes associated with antibiotic targets and virulence genes. For example, using Mobile-CRISPRi-seq technology, a conditional essential gene knockdown library for *K. pneumoniae* was constructed. Combined with high-throughput sequencing, *yciS* and *ribB* were shown to be virulence-related genes that contribute to the pathogenicity of *K. pneumoniae* [89].

## 5. Challenges and Future Prospects of the CRISPR-Cas System

Despite the significant advantages of CRISPR-Cas technology in the detection and treatment of resistant bacteria, it still faces some challenges. The first challenge is the issue of gene editing efficiency and delivery efficiency. Homology-directed repair (HDR) results in low efficiency, and CRISPR knockouts may induce gene compensation reactions, which sometimes prevent the model from showing the expected phenotypic differences [90]. The mainstream CRISPR-Cas delivery methods currently include phages, plasmids, and nanoparticles. The suitability of different delivery approaches varies considerably for bloodstream, pulmonary, and intestinal colonization scenarios. As summarized earlier, bacteriophages are naturally suited for targeted delivery in bloodstream and gut colonization scenarios, where they can specifically infect *Klebsiella pneumoniae* strains. However, their narrow host range and strain-specificity limit their broad application across different CRKP lineages [91]. Plasmid delivery, relying on electroporation or co-culture, is currently limited to in vitro and preclinical ex vivo models, making it impractical for systemic in vivo use, especially in bloodstream infections [92]. Nanoparticle delivery, by contrast, offers greater versatility for intravenous or aerosol administration, which is more relevant for bloodstream and pulmonary infections, though its in vivo stability and biocompatibility remain key hurdles to overcome [93,94]. The second issue to focus on is off-target effects, especially with Cas12a and Cas9, which may induce large fragment deletions and chromatin fragmentation at non-target sites. This not only affects the precision of gene editing but could also bring potential toxic side effects, such as disrupting the human microbiome and disturbing the microecological balance [95,96]. Notably, resistance genes differ substantially between plasmid-encoded and chromosomal loci. Many of the carbapenemase genes discussed earlier (e.g., *KPC*, *NDM*, *OXA-48*) are plasmid-encoded, which presents both advantages and challenges. Plasmid-borne resistance genes are often present in multiple copies, which can increase editing efficiency, but also raises concerns about the potential for plasmid loss and horizontal transfer. In contrast, targeting chromosomally encoded genes, such as porins or efflux pump regulators, requires more precise and stable delivery to avoid the emergence of escape mutants.

Finally, attention should be given to the long-term in vivo effects after CRISPR knockout, to evaluate whether prolonged expression leads to cytotoxicity and the feasibility of long-term application. Among bacterial defense mechanisms, some bacteria produce anti-CRISPR (Acr) proteins that inhibit the cleavage activity of the CRISPR-Cas system. These proteins exert their inhibitory effects by directly interacting with Cas proteins, thereby disrupting key steps such as target DNA binding and cleavage, crRNA loading, or effector complex formation. As a result, Acr proteins effectively suppress CRISPR-Cas-mediated gene editing [97]. This phenomenon carries dual implications. On one hand, the presence of Acr proteins highlights the evolutionary adaptability of bacteria and underscores the need to consider potential resistance mechanisms when employing CRISPR-Cas systems for therapeutic purposes. Overcoming such resistance will be essential to maintain the long-term efficacy of CRISPR-based treatments [98]. On the other hand, the ability of Acr proteins to modulate CRISPR-Cas activity offers a powerful tool for genetically encoded post-translational regulation. This can enhance the precision of gene editing applications [83]. In both in vivo and ex vivo contexts, uncontrolled Cas nuclease activity may lead to adverse outcomes such as off-target effects, cytotoxicity, or immunogenicity [99,100]. Acr proteins can serve as regulatory elements to mitigate these risks, offering a means to fine-tune CRISPR-Cas function and improve the safety profile of gene editing strategies. We should take advantage of this property of Acr to improve the accuracy of the CRISPR-Cas gene editing function.

Clinical translation of CRISPR-Cas technology also faces multiple unresolved prerequisites. To address and resolve these challenges, future research directions should focus on the following areas. First, the development of more efficient delivery systems is necessary. With advancements in science and technology, the development of new types of nanoparticles and biosynthetic carriers may help improve the delivery efficiency of CRISPR-Cas, making it more suitable for clinical applications [94,101]. Jia have used biomimetic cationic hybrid vesicles (BCVs), which integrate bacterial outer membrane vesicles with cationic lipids, as carriers for Cas9, achieving efficient in vivo and in vitro delivery of the CRISPR system [102], The newly developed circular carriers can replace plasmids in cell delivery, offering higher efficiency and reduced cytotoxicity by not containing the cell skeleton [103]. Next, by optimizing sgRNA design and engineering Cas proteins, such as exploring smaller and more efficient Cas enzymes like Cas14, could improve the precision of gene editing and reduce off-target effects [12,93,104,105,106]. In addition to other necessary conditions, combining the CRISPR-Cas system with traditional antibiotics or phage therapy could enhance therapeutic effects and reduce the survival rate of resistant bacteria [107]. Further in vivo clinical trials to assess the safety and efficacy of CRISPR-Cas technology in treating resistant bacterial infections are key prerequisites for its clinical application and translation.

Of course, in terms of the transmission of CRKP resistance, in addition to further realizing the clinical application of CRISPR-Cas in treatment, we should also focus on and conduct real-time resistance monitoring. This is the essential prerequisite for preventing the development and spread of resistant bacteria. Currently, CRISPR-based nanosensor technology can be used to achieve real-time monitoring of resistant bacteria, providing decision-making support for precise medication and fundamentally preventing antibiotic misuse, thus reducing the emergence of resistant bacteria [12].

Beyond the technical and translational challenges, CRISPR-Cas-based antibacterial interventions also raise substantial biosafety and ethical concerns in clinical applications. First, unintended off-target effects on the human commensal microbiota may disrupt microbial homeostasis and potentially influence host immune and metabolic functions. Second, delivery vehicles carrying gene-editing components, such as plasmids, bacteriophages, or other vectors, may undergo horizontal gene transfer within complex microbial environments, thereby facilitating the dissemination of exogenous genetic elements across bacterial populations and increasing ecological risks. In addition, sustained selective pressure may further drive bacterial adaptive evolution, enabling pathogens to evade CRISPR-Cas-mediated killing through mechanisms such as target-site mutations, production of anti-CRISPR factors, or enhanced biofilm formation, ultimately compromising long-term therapeutic efficacy [90,99,100].

Therefore, before the widespread clinical implementation of CRISPR-Cas-based strategies against CRKP infections, comprehensive and rigorous safety evaluation systems must be established, including assessments of off-target activity, microbiome alterations, horizontal gene dissemination, and long-term evolutionary risks. Meanwhile, standardized ethical guidelines, harmonized clinical trial frameworks, and well-defined regulatory mechanisms are urgently needed to balance technological innovation with patient safety, ecological security, and public health interests. Under appropriate ethical oversight and regulatory governance, CRISPR-Cas technology may ultimately achieve safe, standardized, and sustainable clinical translation for the treatment of CRKP infections.

## 6. Conclusions and Prospect

The CRISPR-Cas bacterial immune system has shown significant advantages in the field of gene editing in recent years, with an increasing number of studies applying it to molecular biology, genome engineering, and translational applications [58]. CRKP is a strain that severely impacts global public health, and its resistance can spread worldwide through plasmid-mediated horizontal transfer [27]. Most traditional antibiotics are ineffective against CRKP and the resistance to novel antibiotics is also on the rise year by year [40]. As a revolutionary gene editing tool, the CRISPR-Cas system provides new possibilities for the detection and treatment of bacterial resistance [108]. By combining isothermal amplification techniques and lateral flow immunoassay strips, resistance genes of CRKP can be detected and visualized in a short period [49,57]. This CRISPR-Cas-based detection can assist in the selection of antibiotics for clinical *K. pneumoniae* infections. In addition to resistance gene screening, the CRISPR-Cas system can also be delivered into resistant *K. pneumoniae* through bacteriophages, conjugated plasmids, or nanoparticles, enabling targeted editing of resistance genes and restoring sensitivity to certain antibiotics, providing a novel approach for the clinical treatment of CRKP [109].

In the treatment and study of drug-resistant bacteria, conventional gene editing technologies—such as homologous recombination, zinc finger nucleases (ZFNs), and transcription activator-like effector nucleases (TALENs)—have been employed to manipulate bacterial genomes. However, these methods are often limited by technical complexity, low editing efficiency, and a high risk of off-target effects [110,111]. In contrast, the CRISPR-Cas system offers significant advantages in terms of precision, efficiency, scalability, and ease of use, making it a superior tool for investigating bacterial virulence factors and antimicrobial resistance mechanisms [112]. A key feature of the CRISPR-Cas system is its ability to achieve targeted genome editing guided by a single guide RNA (sgRNA), which directs the Cas nuclease to a specific DNA sequence with high specificity. This targeted approach enables efficient and precise editing of specific gene sequence, facilitating the dissection of complex genetic networks and the simultaneous manipulation of multiple resistance genes [13,14]. Moreover, unlike traditional gene editing tools, the CRISPR-Cas system does not require elaborate homology arm design or multi-step screening processes, significantly streamlining experimental workflows and increasing editing throughput. Its modularity and programmability allow for the rapid re-design of sgRNAs to target different genes without the need for specialized restriction enzymes, thereby broadening its applicability across diverse bacterial species and genetic contexts [112]. In summary, the CRISPR-Cas system represents a next-generation gene editing technology that overcomes many of the limitations of earlier tools. Its precision, efficiency, and flexibility make it especially well-suited for advancing research on drug resistance and for developing novel antimicrobial strategies.

Although CRISPR-Cas technology is still in the research stage, its potential in the treatment of drug-resistant bacterial infections cannot be ignored. Current studies have already demonstrated several advantages of the CRISPR-Cas system in treating resistant bacteria, but its practical application is still limited by factors such as delivery efficiency, off-target effects, and bacterial anti-CRISPR defense mechanisms [113]. In order to achieve widespread clinical application, there is still a long way to go for CRISPR-Cas technology. With the advancement of science and technology, focusing on optimizing delivery systems, improving targeting accuracy, reducing off-target effects, and addressing bacterial anti-CRISPR mechanisms will be important breakthroughs. For example, expanding the Cas9 domain or using smaller nucleases can enhance base editing accuracy and reduce off-target efficiency [114]. With the gradual progress and maturation of biotechnology and nanotechnology, the CRISPR-Cas system is expected to provide better solutions for the detection and treatment of drug-resistant bacterial infections in the future, becoming a crucial tool for precision medicine.

## Figures and Tables

**Table 1:** Introduction to the CRISPR-Cas System.

CRISPR-Cas System	Type	Domain	Target Site	DNA/RMA Recognition	PAM Requirement	Mechanism of Action	References
Cas12a	Type V	RuvC	ssDNA dsDNA	crRNA	5′-TTN-3′	Cleavage of dsDNA, leading to single-strand DNA degradation	[10]
Cas13a	Type VI	HEPN	ssRNA	crRNA	NO	RNA-mediated targeted RNA degradation	[11]
Cas14a	Type V	HEPN, REC, NUC	ssDNA	sgRNA	NO	Mainly used for high-specificity DNA recognition and cleavage	[12]
Cas9	Type II	RuvC, NHN	dsDNA	sgRNA	5′-NGG-3′	Induce dsDNA break	[13]
Cas3	Type I	HD, RecBCD	dsDNA	crRNA	Need to match the PAM sequence	Possesses nuclease exonuclease activity and can degrade large fragments of DNA	[14]
CRISPRi	Type II derivative	Cas9 (inactive dCas9)	dsDNA	sgRNA	5′-NGG-3′	Inhibit gene expression by blocking RNA polymerase binding to DNA	[15]

**Table 2:** Application of CRISPR-CAS System in CRKP Detection.

Detection Technology	Target Gene	Sample Type	LOD (Original Unit)	Sensitivity (%)	Specificity (%)	Accuracy (%)	Reading Method	References
LAMP-Cas12a	*KPC*, *NDM*	Spiked sample	≥3 × 10^5^ CFU/mL	100	100	100	LFS	[44]
RPA-Cas12a	*KPC*, *NDM*	Clinical isolates	1 aM (plasmid)/5 × 10^3^ fg/μL (gDNA)	NR	NR	100	LFS, FD	[45]
Dual RPA-Cas12a	*rcsA*, *KPC*	Clinical isolates	*KPC*: 10 fg/μL; *NDM*: 1 ng/μL	100 (*rcsA*)/85.71 (*KPC*)	100 (both)	100/94.92	FD, BLID, UID, LFS	[46]
RPA-Cas12a	*KPC*	Clinical isolates	*rcsA*: 1 pg/μL (FD); *KPC*: 10^2^ pg/μL	100	100	100	LFS, FD	[47]
PCR/RAA-Cas13a	*KP*, *KPC*, *NDM*	Clinical isolates	1 copy/μL;10^1^ copies/μL (RAA)/1 CFU/cm^2^ (swab)	100 (each target)	100	92.16 (CRKP positivity)	LFS, FD	[48]
RPA-Cas13a	*KPC*	Clinical isolates, surface swabs	2.5 copies/μL	96.5	100	100	FD	[49]
LAMP-Cas13a	*OXA-48*, *GES-2*	Clinical isolates, sputum	10^3^ CFU/mL	100	100	100	LFS	[50]

Abbreviations: a LOD units vary across studies (CFU/mL, aM, fg/μL, pg/μL, ng/μL, copies/μL) and cannot be directly converted without unrealistic assumptions. Direct numerical comparison between rows with different units is not recommended. b FD = fluorescence detection; BLID = blue light irradiation detection; UID = ultraviolet irradiation detection; LFS = lateral flow strip; NR = not reported. c Accuracy for [45,46] refers to the positive agreement rate for CRKP detection compared to reference methods; for others, accuracy is as reported in the original study.

## Data Availability

No new data were created or analyzed in this study. Data sharing is not applicable to this article.
